# Qualitative Exploration of the Suitability of Capability Based Instruments to Measure Quality of Life in Family Carers of People with Dementia

**DOI:** 10.1155/2014/919613

**Published:** 2014-03-04

**Authors:** Carys Jones, Rhiannon Tudor Edwards, Barry Hounsome

**Affiliations:** ^1^Centre for Health Economics and Medicines Evaluation, Bangor University, Ardudwy Hall, Bangor LL57 2PZ, UK; ^2^Faculty of Health Sciences, University of Southampton, Highfield, Southampton SO17 1BJ, UK

## Abstract

*Background*. In an ageing population, many individuals find themselves becoming a carer for an elderly relative. This qualitative study explores aspects of quality of life affected by caring for a person with dementia, with the aim of identifying whether capability based questionnaires are suitable for measuring carer quality of life. *Methods*. Semistructured interviews lasting up to an hour were conducted, November 2010–July 2011, with eight family carers of people with dementia. Interviews typically took place at the participants' homes and were recorded and transcribed verbatim. Framework analysis was used to code and analyse data. Domains from three capability based questionnaires (ICECAP-O, Carer Experience Scale, and ASCOT) were used as initial codes. Similar codes were grouped into categories, and broader themes were developed from these categories. *Results*. Four themes were identified: social network and relationships; interactions with agencies; recognition of role; and time for oneself. *Conclusions*. By identifying what affects carers' quality of life, an appropriate choice can be made when selecting instruments for future carer research. The themes identified had a high degree of overlap with the capability instruments, suggesting that the capabilities approach would be suitable for future research involving carers of people with dementia.

## 1. Introduction

Dementia is a growing problem, affecting over 800,000 people in the United Kingdom at an annual cost of *£*23 billion [1]. Informal care by friends and family accounts for 55% (*£*12bn) of this cost [2]; this would have to be covered by health and social services, if carers were unable to cope. The number affected by dementia is expected to rise with the ageing population, placing a heavier burden on both families and health and social services in the future. From an economic perspective, it is important to support informal carers in their role to enable people with dementia to remain living at home as long as possible. In response to the increasing pressure being placed on scarce health and social care resources, the UK government has made a commitment to double spending on dementia research to *£*66 million per year by 2015 following calls by the Alzheimer's Society to increase funding substantially [3]; therefore, it is timely to consider the appropriateness of current health economics instruments used to measure quality of life.

In a survey of the general UK population, respondents were asked to list up to five things that affected quality of life. Over 60% stated relationships with family and other people, 43% selected their own health, and 38% selected the health of somebody they were close to [4]. Caring has been found to affect mental health more than physical health [5], with up to 30% of carers of people with dementia experiencing depression [6].

With increased public expectations of what treatments should be made available on the National Health Service (NHS), choices have to be made about whether or not to fund specific health care interventions. Informed funding decisions can only be made after a rigorous economic analysis of the costs and benefits of competing alternatives has taken place. In the UK, the National Institute for Health and Care Excellence (NICE) assesses evidence on the clinical-effectiveness and cost-effectiveness of treatments. The NICE guide to technology appraisal [7] states that effectiveness should be reported in quality-adjusted life years (QALYs), with the EQ-5D [8] their preferred questionnaire for measuring health-related quality of life component of the QALY. Guidance on whether the NHS should fund a treatment is based on whether the cost per QALY falls below an arbitrarily chosen funding threshold of *£*20,000–*£*30,000.

However, despite NICE favouring the use of the EQ-5D, there are arguments for including alternative outcome measures. The EQ-5D is dominated by physical health questions, which places a “patient's” identity on the carer [9]. Carer interventions can cross the health and social care sector; therefore, instruments that focus on physical functioning underestimate the full effects on quality of life. In this paper, we argue for the routine inclusion of broader quality of life measures alongside the EQ-5D in research involving carers of people with dementia.

Capability theory is a growing area in health economics. Recent developments include three instruments: the ICECAP-O [10], the Carer Experience Scale (CES) [11, 12], and the Adult Social Care Outcomes Toolkit (ASCOT) [13]. The ICECAP-O was developed through prior research into quality of life of members of the general population aged 65+. It is an appropriate instrument as many carers of people with dementia fall into this age group. The ICECAP-O has been validated for use with the general older population and in a hospital setting [14, 15]. The Carer Experience Scale was developed through qualitative research with carers and validated through a survey of carers in the general population [16]. The ASCOT was developed to measure social care related quality of life for the care recipient but the domains might be applicable for evaluation of quality of life for carers too. The ASCOT has been validated for use with older people [17]. While similar in format to the EQ-5D, these instruments contain domains which measure the capability of an individual to achieve a range of outcomes. To explore whether these instruments would be suitable for use with carers of people with dementia, a qualitative approach was adopted. Qualitative research allows a deeper exploration of a subject and can be conducted alongside quantitative research to enhance understanding and put results into a meaningful context [18]. While it is not often possible to generalise findings across a whole population, qualitative research is a useful tool for informing the choice of instruments used in quantitative methods. The qualitative research described in this paper was undertaken to explore the question “what do family carers of people with dementia perceive as affecting quality of life and how well do capability based instruments capture these aspects of quality of life?”

## 2. Methods

### 2.1. Design

A framework analysis approach was used to analyse the data. Framework analysis was selected as it is systematic and allows transparency in the data analysis [19]. The framework approach is popular in healthcare research. It is the opposite of more inductive approaches, such as grounded theory, as the focus is not on developing a new theory but instead on describing and interpreting participants' views. The COREQ checklist [20] is used to report the qualitative research presented in this paper.

### 2.2. Participants and Recruitment

Carers were recruited through distributing information sheets face-to-face at Alzheimer's Cafes in North Wales and through the mailing list of the NEURODEM (Wales Dementias and Neurodegenerative Diseases Research Network) Research Participant Register, a register of carers and people with memory problems who have given permission to be contacted about research projects. The information sheet explained the aim of the study, and that information was being collected as part of a PhD study examining quality of life measurement in carers of people with dementia. Carers were asked to contact the lead author (Carys Jones), if they were interested in participating. Carys Jones did not know any of the participants prior to recruitment. It did not matter whether participants were current or former carers because a “lived experience” viewpoint was sought. Convenience sampling was used and participants were selected opportunistically to ensure as homogeneous a sample as possible. No target sample size was set; participants were recruited until data saturation occurred.

### 2.3. Interview Procedure

Due to the potentially sensitive nature of the topic, one-to-one interviews were held rather than focus groups. Semistructured interviews were held at a location convenient to the carer, typically their home, between November 2010 and July 2011. Interviews were planned to be held with only the interviewer and participant present; however, in two of the interviews the person with dementia was also present. Interviews were conducted by the lead author (Carys Jones), a female PhD student. Before the interview commenced, Carys Jones asked participants to read the information sheet again and provide written consent to take part. Participants were reminded that they could stop the interview or ask questions at any time. Repeat interviews were not conducted.

An interview schedule containing open ended questions about the participant's experiences as a carer was used. Questions were designed to encourage participants to talk about both past experiences and concerns about the future. Prompts were used to encourage the participant to elaborate more on topics. The interview schedule was not tested prior to use; however, after each interview, the schedule was reviewed to determine whether modifications were needed. Interviews lasted between 22 and 54 minutes and were recorded using a digital recorder, with additional notes taken during the interview.

### 2.4. Data Analysis

Interviews were transcribed verbatim by Carys Jones with identifying information, such as names, changed to protect confidentiality. Analysis was undertaken in QSR International's NVivo 8 qualitative data analysis software [21].

The lead author familiarised herself with data through repeated listening to the recordings and reading of all of the transcripts. The domains of the EQ-5D, ICECAP-O, CES, and ASCOT were used as predetermined codes. The ICECAP-O measures an individual's capability to achieve an outcome regardless of whether they carry out the functioning; for example, respondents are asked whether they *can* have all the love and friendship that they want rather than if they *have* all the love and friendship that they want [10]. The domains of the ICECAP-O are love and friendship, thinking about the future, doing things that make you feel valued, enjoyment/pleasure, and independence. There are four possible levels for each domain: I can have all, I can have a lot, I can have a little, and I cannot have any. The ICECAP-O is scored from 0 (no capability) to 1 (full capability). The CES covers both the positive and negative aspects of caring. Its six domains are activities outside caring, support from family and friends, assistance from organisations and the Government, fulfilment from caring, control over caring, and getting on with the person you care for. Each domain has three possible levels: a lot, some, and little. The CES is scored from 0 (worst caring state) to 100 (best caring state). Domains of the ASCOT are control over daily life; personal cleanliness and comfort; food and drink; personal safety; social participation and involvement; occupation; accommodation cleanliness and comfort; and dignity. Each domain has four levels and the ASCOT is scored between −0.17 (no care needs met) and 1 (all care needs met).

The predetermined codes (i.e., the domains of the capability instruments) were sought in the data using a line by line coding method in NVivo by Carys Jones. Additional codes were derived inductively. A sample of transcripts was reviewed by coauthors Rhiannon Tudor Edwards and Barry Hounsome to improve rigour; however, as Carys Jones had led the research and conducted the interviews, she was more immersed in the data and was ultimately responsible for coding decisions. Similar ideas thought to affect quality of life were grouped into categories, which were then refined into broader themes. The original transcripts were cross-checked to ensure that the themes and their interpretation were grounded in the participant's descriptions. Negative cases were sought to identify contradictions.

Quotes presented in the text were selected for clarity and relevance. Sections not relevant to the theme have been removed and replaced by ellipses (…). Repetition and hesitations not thought to add meaning, such as “erm,” “you know,” and “umm”, have been removed without ellipses.

### 2.5. Data Protection

In compliance with the terms of the Data Protection Act [22], contact details for participants were stored securely in a password protected file on a computer that only Carys Jones had access to. Anonymised transcripts were also stored securely on the computer. Hard copies of consent forms were archived in a locked cabinet in a locked room, the key being held by Carys Jones.

### 2.6. Ethical Approval

Ethical approval for the study was received from Bangor University.

## 3. Results

Eight carers were recruited; participant characteristics are displayed in Table 1. Four themes were identified: social network and relationships; interactions with agencies; recognition of role; and time for oneself.  Table 2 shows the initial codes identified, along with the resulting broader themes.

### 3.1. Social Network and Relationships

This theme encompasses the social support that carers perceive they have and how their relationships with both the person with dementia and others had changed. Spousal carers looked first to their husband/wife for social support. A change in the ability to communicate with the person being cared for was a source of upset.

 We always had this very strong relationship and we always used to think the same things…Once the Alzheimer's started all his personality changed, that all went, as if we weren't on the same wavelength at all. (C1; female, bereaved spouse).

In the case of the child carer, as dementia had progressed, it facilitated a closer relationship than had been experienced before.

 I had my arm around her and I always try and massage her back or just touch her hand or just try and be quite tactile with her. And I was thinking, my god she would have hated this.… She wasn't a very tactile person at all… it's come to it…that this has got to happen for us to actually give her a hug. (C4; female, parent in long-term care).

Family and friends were seen as a secondary social support network, both for practical care tasks and emotional support.

 We've got two very good sons that live close by…one doesn't do much with his dad but he'll come and say to me “Oh tidy yourself up and I'll take you out for a meal”.…The other son, we see him nearly every day, and he does what his dad can't do anymore. He mends things… Listening to a lot of people, I think I'm alright. (C6; female, spouse).

Relationships with friends and family could also become strained, if there was a lack of understanding about dementia.

 He came from a big family…they used to come here and the first questions they used to ask him “do you remember how we used to do so and so?” In the end I had to tell them not to remind him, or ask him questions. Because you'd see then that Robert would get quite frustrated. He wasn't able to remember these things. (C2; female, bereaved spouse).
 They're not interested, won't listen to you. It's family, recently I've been trying to get through (to) them there's a problem but they're not interested, as far as they're concerned you look fine so you are fine. (C7; male, spouse).

Carers were anxious about socialising in a wider circle, if they felt that the behaviour of the person with dementia might cause embarrassment.

 I've been to the memory clinic, or memory café rather, on two or three occasions, but I don't feel that either Brenda or myself have benefitted from that. What it amounts to is that you sit at a table possibly with other people, have a cup of tea and a biscuit, and you might have a talk by the fire brigade or the police, which in the case of Brenda really is of no interest and occasionally she makes adverse remarks very loudly during the lecture, which was an embarrassment. (C8; male, spouse).

Social activities, such as dining out or shopping, were restricted, if it was felt that the person with dementia was not enjoying the experience.

 He'd become very agitated if he was somewhere strange, and with strange people. So I never stayed long. We used to go to the Christmas dinner, but he always wanted to come home. He was quite safe in his own surroundings. (C2; female, bereaved spouse).

### 3.2. Interactions with Agencies

This theme refers to the carers' perceptions about their experiences with medical staff, social services, and organisations such as Crossroads and the Alzheimer's Society. All carers spoke of difficulty in getting a dementia diagnosis. This was typically caused by the person with dementia not acknowledging that there was a problem and refusing to see a doctor or, once an appointment was made, the doctor not confirming the symptoms as being dementia. The lengthy process of getting a diagnosis caused stress and self-questioning about whether there really was something wrong with the person being cared for.

 Naturally for self preservation *[sic]* reasons, clinicians, doctors are very reluctant to say that the patient has Alzheimer's disease. They will mention all sorts of things without actually saying it. (C8; male, spouse).
 It took a year to get the diagnosis. Which I think is probably actually fairly quick compared to some people. But it was actually almost a bit of a relief to actually know that we weren't sort of imagining that it *[sic]*. (C3; female, spouse).

Carers felt that information received from various agencies was fragmented and not received at an appropriate stage of the illness. Some stated that they would have liked more information at the time of diagnosis; others mentioned that at the start of the transition from spouse to carer they did not want to hear about practical care tasks that might become necessary as the person with dementia deteriorated, such as dealing with incontinence and feeding needs. Some carers also found the amount of extra support received immediately after a dementia diagnosis to be overwhelming.

 We had all kinds of people come in.…They sent people in to put ramps. I had ramps everywhere in this house. Outside, inside, everything. They put fire alarms in. The physio *[sic]* came. A social worker came…. Somebody else came to see if we had enough benefits…Constant, constant visitors. Perhaps it was a bit much, but….they were trying to help. (C1; female, bereaved spouse).

Two carers mentioned that it was difficult to access health and social care services during the night-time. One was reluctant to use “out of hours” services unless it was an emergency situation as he did not want to be seen as a burden.

 The CPN gave good support and she gave good advice all along. Occasionally I refused it because I thought I could go my own way, but I was in the wrong. She was very supportive; I could even ring her at night… One of the things that was missing in my case,… having any support… at night…The CPN said “You can ring me any time” which wasn't quite true as her mobile would be turned off because she was tending someone else or something else, and at night time I did not like calling because it's her time off. (C5; male, spouse in long-term care).

Where services had not met the carer's expectations, a sense of cynicism was harboured.

 It got to a stage where it was 2 o'clock in the afternoon and she was still in bed, and I felt pretty desperate about it. I got in touch with the social services that suggested that perhaps if someone came in, she would respond to a figure of authority…They used to come in for perhaps ten or fifteen minutes, if she would still not get up they said because they're not allowed to physically intervene they would come and ask for my help anyway. I felt that was a bit of a fiasco. At the end of the couple of months or whatever I had a bill for *£*300, and I didn't really feel that I'd had very much in the way of assistance. (C8; male, spouse).
 Whenever I went to the Alzheimer's Society to ask for advice they say “Oh, we're not at liberty to give out specific advice” like *[sic]* which homes shall I go to. … The CPN, community health care people, they said the same: “We're not allowed to recommend homes.” (C5; male, spouse in long-term care).

### 3.3. Recognition of Role

Recognition of role was perceived as an important theme. Caring can be associated with increased levels of stress and depression; however, positive aspects such as fulfilment from caring were identified.

 In the past Charles would have dealt with a lot of the things that I now…may be don't deal with but I help him with… He would have dealt with all the financial side of things and the paying of the bills and although I drove, he used to like to do a lot of the driving.….He wasn't a great DIY person or anything like that but it's all the sort of small things that you don't really think that he automatically used to do that I now find myself doing. So I'm probably busier than I have been for quite a few years (laughing). (C3; female, spouse).

Seven participants mentioned events which they believe triggered or accelerated the onset of dementia in the person they cared for. Carers felt guilt or blamed themselves for those events even if they were unavoidable, such as having their own health problems.

 When I had the operation I was away from her for two weeks ‘cause I had the operation in Cambridge and she had to look after the dogs at home. So she didn't see me for two weeks and when I came back she was quite distressed. She was quite agitated. (C5; male, spouse in long-term care).

All of the carers had successfully adapted to the change in role from being a spouse/child to a carer.

 I'm very much like “Right, ok, things happen, I need to work on it. I need to be positive and find out more and do things.” I just tend to react to things like that a little bit I think. (C4; female, parent in long-term care).

Carers were keen to help others through recording their own experiences and raising awareness of dementia and its progression over time.

 And I do try and talk to people that I know are going through it, right at the beginning and try and give as much of my information as I can, the things that we went through. That's why I'd quite like to get this little diary that I've got published as a little book.… I just think you could go through and go “God, that's normal, that bit that happened.” (C4; female, parent in long-term care).

### 3.4. Time for Oneself

The theme of time for oneself recognises the perceived value of having time away from the person being cared for and hence time away from caring. Participants spoke of being able to resume activities they had previously discontinued because the person being cared for had not shared their interest.

 This sounds strange; I've got my life back. My wife and I were opposites when we met…At the time I thought this might be a good marriage, because we can then each benefit from each other's experience. But it never really worked out like that; I tended to abandon all my academic interests… I didn't begrudge at the time, but now that she's off, and I have every other day to myself and doing things which I did in my youth…I'm taking up movie making again, and things like this, which I did before we got married. (C5; male, spouse in long-term care).

A sense of frustration was evident when the carer was not able to spend time away from the person being cared for.

 Television now has become just action; all he can watch is these action things like Schwarzenegger…I can't stand them but I've got to sometimes go along with it and try and read…We do have two televisions at home but if I go to watch he'll find (me) and say “What you watching? I think I'll watch that then” ‘cause he wants to be with me. That's awkward. (C6; female, spouse).

As well as having a greater feeling of independence, the carers of people who had moved into long-term care also spoke of the guilt they experienced at having to make the decision.

 It's almost like having rent-a-wife…It's awful, in one way it gives me the freedom but on the other hand I feel awful picking her up for a little while, you know, having a good time and then just dumping her. (C5; male, spouse in long-term care).

## 4. Discussion

Interventions involving carers of people with dementia might have multiple objectives, such as improving burden, coping skills, and general quality of life. The need to select appropriate outcome measures for economic evaluations has been recognised [23, 24]. By focusing on health-related quality of life measurement, the NICE guide to technology appraisal [7] can overlook nonphysical benefits of interventions. Bodies allocating research funding should check that outcome measures listed for proposed research match the objectives of the intervention rather than relying on the use of historically popular measures. The capability instruments that we choose to explore are validated and can be easily completed by older people. There is scope to improve current research practice by considering these alternative measures to capture quality of life. In the context of family medicine, using instruments which are sensitive enough to detect subtle changes in quality of life will lead to more informed decisions being made when scarce health care resources are being allocated.

As Coast discussed [18], there is a role for qualitative work in the traditionally quantitative field of health economics; however, researchers must be careful to use methods appropriately to produce work which passes the scrutiny of both health economists and qualitative researchers. The aim of this study was to elicit what carers of people with dementia perceived as impacting on their quality of life, and hence whether capability based instruments capture these aspects of quality of life and are appropriate for use in future health services and health economics research. Four themes were identified: social network and relationships; interactions with agencies; recognition of role; and time for oneself.

### 4.1. Social Network and Relationships

A desire for increased social support was a recurring topic. Spouses felt a sense of loss for the person they used to know and sometimes found it difficult to communicate with the person their spouse had become. The subsequent layer of social support was the wider network of friends and family, reflecting the findings of Etters et al. [25], who in a review of carer burden noted the importance of positive family relationships and support. Etters et al. [25] also found that the closer the kinship to the person being cared for, the higher the level of burden perceived. Carers in this study had experienced a reduction in their extended social support network as a result of avoiding social situations and loss of friends due to normal aging. In a trial of a counselling and support intervention compared to usual care for spouses of people with Alzheimer's disease, it was found that carers who utilised support services were able to keep their partner at home longer than those who did not [26]. The mechanism behind this was thought to be an improvement in response to behavioural problems and increased carer satisfaction with social support, which relates to the theme of social network and relationships found in this study. The social network and relationships theme overlapped with all three capabilities based instruments, with the CES exhibiting the most overlap. Only the anxiety/depression domain of the EQ-5D was thought to have a clear conceptual overlap with the social network and relationships theme.

### 4.2. Interactions with Agencies

Interactions with agencies were closely tied to the domain of “assistance from organisations and the government” on the CES. Consistent with the findings of Livingston et al. [27], the diagnosis process was a source of frustration, sometimes caused by the person with dementia refusing to admit to having problems with their memory, and sometimes caused by the medical professional not being supportive. In some cases, the person with dementia appeared to be fine during an appointment so the staff would only assess the symptoms of dementia (or lack of them) that they witnessed themselves. Previous qualitative work involving practitioners revealed four obstacles that delayed a formal diagnosis; therapeutic nihilism; risk avoidance; concerns about self-competency in managing dementia care; and availability of resources [28]. In our study the carers perceived the delay in diagnosis to be mainly concerned with risk avoidance and therapeutic nihilism on the part of the professionals. Under the Carers (Equal Opportunities) Act 2004 [29], carers are entitled to an assessment of their needs by the social services. If there is a delay in diagnosis, it will hold up the process of the carer being assessed and supported. Opinions were mixed about whether carers should be given a lot of information at the time of diagnosis or whether knowing the potential outcomes associated with dementia would be distressing. Having more knowledge about dementia has previously been linked to family carers displaying an increased preference for moving the person with dementia into a long-term care facility [30]. The authors of the study hypothesized that people who were more aware of the potential decline associated with dementia were able to recognize that they might not be able to cope with caring. In our study, carers praised staff who they thought had given them good practical advice and information about caring. These findings suggest that the level of information offered to carers should be judged on a case by case basis, with further information given freely if requested.

### 4.3. Recognition of Role

The biggest change in role experienced by carers in our study occurred for the daughter who was looking after her mother. She described a reversal of roles, where she now assumed the parent role and her mother had regressed to being like a child. Other carers compared the experience to looking after a child, a perception also found in the Quinn et al.'s review of dementia caring [31]. Quinn et al.also found that those with a close emotional relationship with the person being cared for prior to the commencement of caring had lower levels of burden and saw caring as rewarding [31]. As well as describing stress and burden, carers spoke of positive experiences arising from their new role. These ranged from closer relationships between the carer and the person being cared for to a sense of feeling appreciated. One bereaved carer had become a volunteer befriender to continue giving in a carer role. In this study, the carers of people in long-term care had become involved in fundraising and raising awareness of dementia as a way of helping others. Recognition of role is reflected in the control over caring domain of the CES, the occupation domain of the ASCOT and the doing things that make you feel valued domain of the ICECAP-O. The domains of the EQ-5D were not thought to describe this theme well.

### 4.4. Time for Oneself

As well as experiencing fulfilment from caring, participants acknowledged a need for time away from caring. This time was used to catch up on chores as well as pursuing leisure activities. The ability to be independent depends on the level of support received from the social network and agencies so this theme is closely linked to the first two. Younger carers often have more competing time demands as they juggle caring, working, and looking after their own family [31], and this was found to be the case for the child carer included in our study. However, a review of caregiver burden and depression suggested that adult child caregivers are more likely to have alternative roles and social activities outside of caregiving, which might moderate the stresses associated with caregiving [32]. The ASCOT has three domains that cover the time for oneself theme: control over daily life; accommodation, cleanliness, and comfort; and personal cleanliness and comfort. The ICECAP-O domains of independence and thinking about the future were also linked to the theme. Two themes of the EQ-5D were thought to overlap with the theme: self-care and usual activities.

## 5. Conclusion

Exploring quality of life and how experiences shape the capability of individuals to successfully cope with caring is of great importance in dementia care. The EQ-5D, which focuses on physical health, had the least amount of conceptual overlap with the identified themes. Two EQ-5D domains were not thought to fit in with the quality of life themes identified by this work: pain/discomfort and mobility. As NICE prefers cost-effectiveness to be reported as a cost per QALY, calculated with a preference based utility measure, there is a need to continue using the EQ-5D in research involving carers of people with dementia. The themes emerging from this exploratory qualitative analysis suggest that capability based instruments are a potential addition to the health economists' toolkit for measuring quality of life in carers of people with dementia. All domains of the ICECAP-O and CES were thought to overlap with the identified themes. This is unsurprising as both instruments were designed to measure capability based quality of life in similar populations; the ICECAP-O for an older population; and the CES for carers. The ASCOT had three domains that did not fit with the themes: dignity, food and drink, and personal safety. However, the ASCOT was developed primarily for use with the person receiving social care rather than their carer. For this reason it might be that the ICECAP-O and CES are more suitable for carer research. Both instruments have their strengths and limitations in this area. The ICECAP-O was designed to capture capability based quality of life of people aged 65 years and over; so while it will be suitable for a lot of spousal carers, it will not be appropriate for younger people, such as child carers. Some people do not self-identify as carers, and as such may feel more comfortable filling out a questionnaire such as the ICECAP-O which asks more broadly about quality of life, instead of answering the questions specific to caring that appear in the CES. The CES was developed using interviews with carers for a range of illnesses, and results from this study suggest that it would be equally suitable for dementia carers. In research aiming to capture a greater insight into the pragmatic experience of caring for people with dementia, the CES would be preferable to the ICECAP-O.

*Limitations*. Qualitative work should be interpreted in its context, which restricts the generalisability of results. Carers in this study were recruited through the Alzheimer's Society and a research register. As such, participants were engaged with a number of local services already and might not be representative of families who do not yet have a formal dementia diagnosis. Carers were offered a choice of interview location; in two interviews the person with dementia was present, which might have made the carer uncomfortable discussing the negative impact of caring. All participants were white and living in suburban or semirural locations; it is unclear whether different themes would emerge from the experiences of carers of different ethnicities or living in an urban area with better access to services and this is an area that needs to be explored in future work.

## Figures and Tables

**Table 1:** Characteristics of participants and recipients of their care.

	*N* (%)
Sex of carer	
Female	5 (62.5%)
Male	3 (37.5%)
Relationship to person with dementia	
Spouse	7 (87.5%)
Child	1 (12.5%)
Location of care recipient	
Living with carer	4 (50%)
Long-term residential care	2 (25%)
Deceased	2 (25%)
Carer employment status	
Retired	5 (62.5%)
Long-term sick	1 (12.5%)
Part-time employment	1 (12.5%)
Full-time employment	1 (12.5%)
Mean age of carer*	69.4

*3 carers did not wish to disclose their age.

**Table 2:** Codes, categories, and themes.

Predetermined codes	Inductively derived codes	Category	Theme
Social participation and involvement (ASCOT)	Participation	Long term effects of caring; concern for the future	Social network and relationships
Enjoyment and pleasure (ICECAP-O)	Positive coping		
Love and friendship (ICECAP-O)	Being positive		
Anxiety/depression (EQ-5D)	Blame		
Support from family and friends (CES)	Safety		
Activities outside caring (CES)	Embarrassment		
Getting on with the person you care for (CES)	Isolation		
Fulfilment from caring (CES)			

Assistance from organisations and the government (CES)	Dealing with professionals	Feelings about involvement with decisions; beliefs about health and social care agencies	Interactions with agencies
	Recording experiences		
	Need for information		

Control over caring (CES)	Raising awareness	Helping others	Recognition of role
Doing things that make you feel valued (ICECAP-O)	Respect		
Occupation (ASCOT)	Person with dementia awareness		

Self-care (EQ-5D)	Own health	Direct impact of caring	Time for yourself
Usual activities (EQ-5D)	Feeling overwhelmed		
Independence (ICECAP-O)	Frustration		
Thinking about the future (ICECAP-O)	Guilt		
Control over daily life (CES)	Dignity		
Accommodation, cleanliness, and comfort (CES)	Difficulty articulating		
Personal cleanliness and comfort (CES)			

CES: Carer Experience Scale; ASCOT: Adult Social Care Outcomes Toolkit.
